# Association of Monocyte Subsets with Carotid Artery Stenosis Severity

**DOI:** 10.3390/medicina62091817

**Published:** 2026-09-21

**Authors:** Levent Deniz, Derya Sonmez, Mehmet Semih Cakir, Eren Ozgur, Ozlem Akdogan, Zahide Adiyaman, Hale Aral

**Affiliations:** 1 Department of Medical Biochemistry, Istanbul Training and Research Hospital, University of Health Sciences Türkiye, 34098 Istanbul, Türkiye; deryasonmeztr@hotmail.com; 2 Department of Radiology, Istanbul Medical Faculty, Istanbul University, 34093 Istanbul, Türkiye; mehmetsemihcakir@gmail.com; 3 Department of Radiology, Istanbul Training and Research Hospital, University of Health Sciences Türkiye, 34098 Istanbul, Türkiye; dr.erenozgr@gmail.com; 4 Department of Neurology, Istanbul Training and Research Hospital, University of Health Sciences Türkiye, 34098 Istanbul, Türkiye; drozlemakdogan@gmail.com; 5 Department of Medical Biochemistry, Kilis Public Health Laboratory, 79000 Kilis, Türkiye; zahide2102@gmail.com; 6 Department of Medical Biochemistry, Private Medicana Ataköy Hospital, Istanbul Yeni Yüzyıl University, 34158 Istanbul, Türkiye; drhalearal@yahoo.com

**Keywords:** carotid artery stenosis, monocyte subsets, classical monocytes, non-classical monocytes, carotid intima–media thickness, inflammation

## Abstract

*Background and Objectives*: This study aimed to investigate the association between circulating monocyte subset profiles and carotid artery stenosis (CAS) severity and to examine their relationships with inflammatory and atherosclerosis-related parameters. *Materials and Methods*: This cross-sectional study included 73 adult patients who underwent carotid Doppler ultrasonography. Participants were classified according to internal CAS severity as <50% (n = 44) or ≥50% (n = 29). Monocyte subsets were analyzed using flow cytometry and categorized as classical (CD14++CD16−), intermediate (CD14++CD16+), and non-classical (CD14+CD16++) monocytes. Serum MCP-1 concentrations were measured using an enzyme-linked immunosorbent assay. Clinical characteristics, biochemical parameters, inflammatory indices, and carotid intima–media thickness (cIMT) were compared between the groups. Multivariate logistic regression analyses were performed to identify independent associations with significant stenosis. *Results*: Patients with CAS ≥50% exhibited significantly higher classical monocyte percentages and lower non-classical monocyte percentages than those with CAS < 50% (*p* = 0.044 and *p* = 0.027, respectively). Intermediate monocyte percentages did not differ significantly between the groups. Significant stenosis was also associated with a higher white blood cell count, neutrophil count, systemic inflammatory response index, fibrinogen, and HbA1c levels. Non-classical monocyte percentages showed a significant inverse correlation with cIMT (r = −0.320, *p* = 0.006). In a multivariable model adjusted for age, sex, and systolic blood pressure, lower non-classical monocyte percentage was associated with significant CAS (OR 0.872, 95% CI 0.765–0.994; *p* = 0.040). However, this association was no longer statistically significant after further adjustment for fibrinogen, neutrophil count, and HbA1c, whereas fibrinogen remained independently associated with significant CAS. *Conclusions*: Lower circulating non-classical monocyte percentages were associated with significant CAS and inversely correlated with cIMT and inflammatory markers. However, the attenuation of this association after adjusting for inflammatory parameters suggests that monocyte subset distribution should be interpreted within the broader inflammatory context that accompanies advanced carotid atherosclerosis.

## 1. Introduction

Carotid artery stenosis (CAS) is a major risk factor for ischemic stroke, emphasizing the clinical relevance of identifying and managing this condition [1]. The development of atherosclerosis is primarily linked to the recruitment and activation of monocytes and monocyte-derived macrophages [2,3,4,5]. Monocytes and macrophages are key players in all stages of atherosclerosis, from lesion initiation and expansion to necrosis, rupture, and clinical manifestations of atherosclerosis [6]. Although it has long been known that macrophages play a role in the onset and progression of atherosclerosis, less is known about the involvement of their blood-borne relatives, monocytes, which are represented by several functional subsets [7]. Different monocyte subsets can play distinct roles in the formation, destabilization, and healing of atherosclerotic plaques [2].

Based on the expression of CD14 and CD16, monocytes are typically categorized into classical (CD14++CD16−), intermediate (CD14++CD16+), and non-classical (CD14+CD16++) subsets. Classical monocytes (CD14++CD16−; Mon1) constitute the major subpopulation (about 85%), the intermediate subset (CD14++CD16+; Mon2) makes up about 5%, and non-classical monocytes (CD14+CD16++; Mon3) make up about 10% [8]. These proportions represent the typical distribution reported in healthy individuals and may vary according to the study population and underlying clinical conditions. Subsets of monocytes have been shown to play various roles in different inflammatory conditions, although their precise functions remain debatable [8]. Consequently, circulating monocyte subsets have been extensively investigated in coronary, carotid, and peripheral atherosclerotic diseases, and accumulating evidence suggests that their distribution reflects plaque inflammation and cardiovascular risk [3,4,5]. Zeynalova et al. demonstrated that monocyte subset distributions were significantly associated with cardiovascular risk categories defined by the Framingham Risk Score [9]. Despite these findings, the relationship between circulating monocyte subsets and CAS severity remains incompletely understood, and the available evidence is limited and partly inconsistent [2,7,10]. Furthermore, the associations between monocyte subset distribution, systemic inflammatory markers, and carotid atherosclerotic burden have not been fully elucidated. Understanding the relationship between monocyte subgroups and CAS severity could improve our understanding of the inflammatory mechanisms underlying carotid atherosclerosis.

This study aimed to investigate the association between circulating monocyte subset profiles and CAS severity and to examine their relationships with inflammatory and atherosclerosis-related parameters.

## 2. Materials and Methods

### 2.1. Study Design

This cross-sectional study included patients who were admitted to the Departments of Neurology and Interventional Radiology at the Istanbul Training and Research Hospital between December 2021 and October 2022. Participants were prospectively enrolled during the study period, and peripheral blood samples were collected at enrollment. This study was approved by the Institutional Ethics Committee of Istanbul Training and Research Hospital (Date: 19 November 2021, decision number: 2984). This study was conducted in compliance with the Declaration of Helsinki. Written informed consent was obtained from all participants.

### 2.2. Subjects

Patients aged ≥18 years who underwent carotid Doppler ultrasonography as part of the routine diagnostic evaluation for neurological symptoms suggestive of carotid artery disease were eligible for inclusion. All participants were elective outpatients, and none of the patients presenting to the emergency department were included. Individuals with acute ischemic stroke or transient ischemic attack at presentation were excluded because acute cerebrovascular events may influence the circulating monocyte subset distribution. Additional exclusion criteria included acute infections, acute brain injury, known malignancy, autoimmune disease, chronic inflammatory disease, cardioembolic stroke or embolism of cardiac origin, aortic or other vascular aneurysms/dissection, intracerebral hemorrhage, and a history of immunosuppressive disorders or immunosuppressive therapy.

### 2.3. Carotid Ultrasound Imaging

All ultrasonographic evaluations were performed using the Esaote MyLab™ Seven ultrasonography system (Esaote, Genoa, Italy). B-mode imaging was performed with a linear probe (LA523, 4–13 MHz). Measurements and evaluations were performed by experienced interventional radiologists according to standard protocols. Measurements were taken in the sagittal plane using B-mode imaging. Carotid intima–media thickness (cIMT) measurements were performed manually using electronic calipers on B-mode images. Measurements were obtained in the sagittal plane from the distal 1 cm segment of the common carotid artery, at the posterior wall, as the hyperechoic area between the intima and media layers, selecting a plaque-free segment. Separate measurements were recorded for the right and left carotid arteries, and the mean cIMT was calculated.

The degree of carotid stenosis was determined according to the North American Symptomatic Carotid Endarterectomy Trial (NASCET) criteria. Patients were classified according to the severity of internal carotid artery (ICA) stenosis, as NASCET-based ICA stenosis is the reference standard used for clinical risk stratification and treatment decisions [11]. Stenosis severity was categorized as minimal (0–49%), moderate (50–69%), severe (70–99%), or total occlusion (100%). For statistical analyses, the participants were classified into two groups according to the severity of internal CAS: non-significant stenosis (<50%) and significant stenosis (≥50%).

### 2.4. Laboratory Analysis

Blood samples were centrifuged at 2000× *g* for 10 min to collect serum, which was stored at −80 °C until assayed. Serum monocyte chemoattractant protein-1 (MCP-1) levels were measured using a Human MCP-1 ELISA kit (Catalog no: E-EL-H6005, Elabscience Biotechnology, Houston, TX, USA) in accordance with the manufacturer’s instructions. The measurement range of MCP-1 was 62.5–4000 pg/mL, and the minimum measurable level (sensitivity) was 37.5 pg/mL. The manufacturer reported an intra-assay coefficient of variation of <10%.

Fasting plasma glucose, total cholesterol (TC), triglyceride (TG), low-density lipoprotein cholesterol (LDL-C), high-density lipoprotein cholesterol (HDL-C), remnant cholesterol, non-HDL-C, C-reactive protein (CRP), creatinine, alanine aminotransferase (ALT), gamma-glutamyl transferase (GGT), and albumin levels were measured using a Cobas 8000 analyzer series (Roche Diagnostics, Mannheim, Germany).

Remnant-C was calculated using the following equation:

Remnant-C = TC − LDL-C − HDL-C.

Plasma fibrinogen levels were measured using STA Compact Max (Diagnostica Stago, Asnieres sur Seine, France). Glycated hemoglobin (HbA1c) levels were measured using ADAMS A1c HA8180V (Arkray, Kyoto, Japan). Complete blood count parameters were analyzed using a Mindray BC-6800 automated hematology analyzer (Mindray Bio-Medical Electronics Co., Ltd., Shenzhen, China).

### 2.5. Flow Cytometry Analysis

Whole blood samples were collected using K2 EDTA anticoagulated vacutainer tubes (BD Biosciences, San Jose, CA, USA). Flow cytometric analyses were performed within 1 h of sample collection. Flow cytometric analysis was performed on a 3-laser, 10-color BD FACSLyric flow cytometry analyzer (BD Biosciences, San Jose, CA, USA) and analyzed using BD FACSuite Software (version 1.4; BD Biosciences, San Jose, CA, USA). For staining, 100 μL of whole blood was incubated with a mixture of fluorochrome-conjugated mouse anti-human monoclonal antibodies [5 μL CD14-APC (Clone: MφP9, BD Biosciences, San Jose, CA, USA), 20 μL CD16-FITC (Clone: NKP15, BD Biosciences, San Jose, CA, USA), and 5 μL HLA DR-Pacific Blue (Clone: L243, BioLegend, San Diego, CA, USA)]. Further details of the surface staining procedure are provided in Appendix A.

#### Gating Strategy for Monocyte Subsets

**Figure 1:**
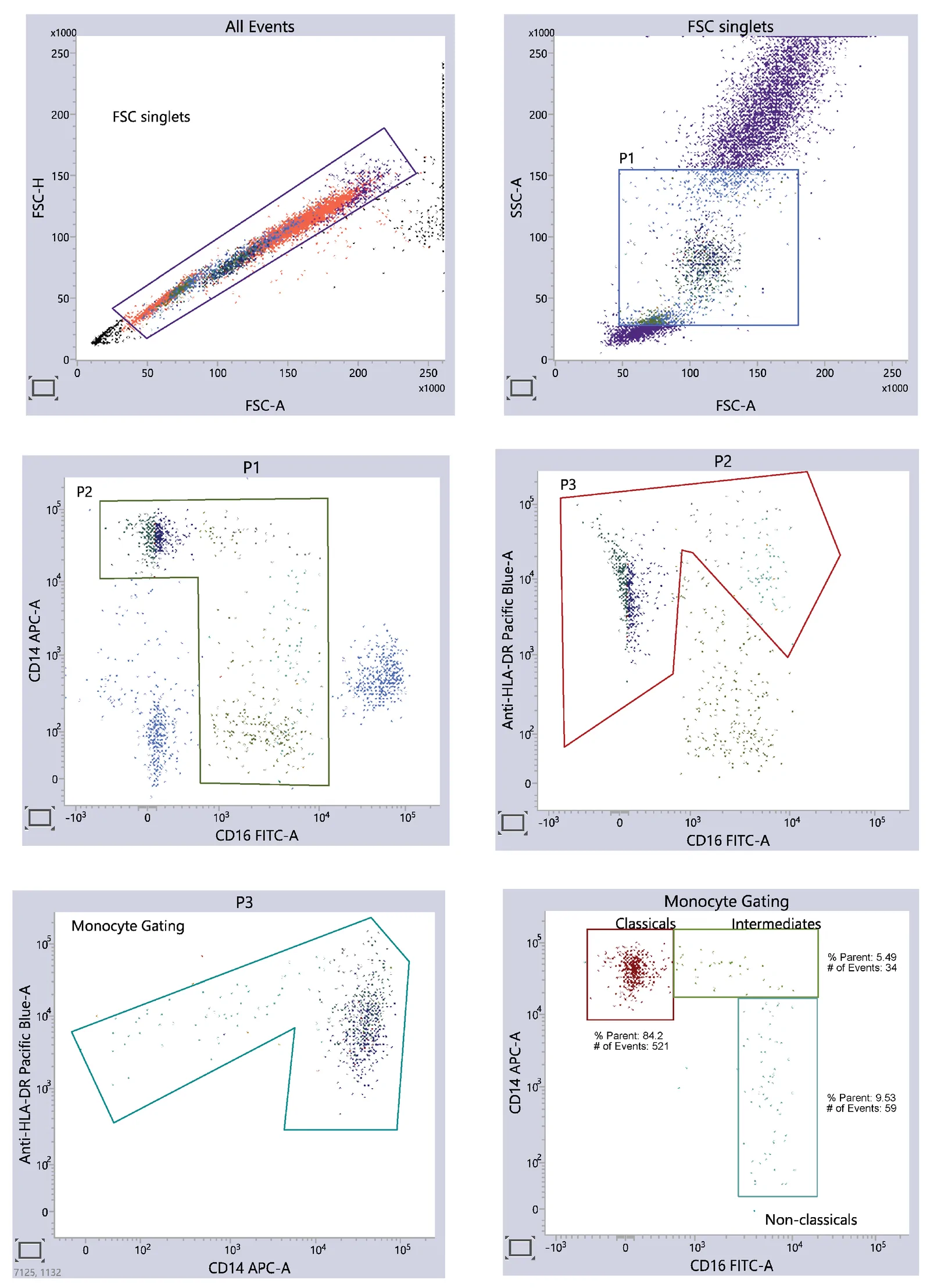
Representative gating strategy for monocyte subset identification.

The gating strategy for monocyte subsets is presented in Figure 1. Initially, doublets were excluded using a forward scatter area (FSC-A) versus forward scatter height (FSC-H) plot. Subsequently, a broad rectangular gate (P1) was established on the FSC-A versus side scatter area (SSC-A) plot to encompass the monocyte population according to its characteristic light-scattering properties while minimizing the exclusion of potential monocyte events [12]. Cells within the P1 gate were subsequently displayed on a CD14 versus CD16 dot plot, and monocytes were identified according to their characteristic “┐”-shaped distribution pattern (P2). To further improve the specificity of monocyte identification, human leukocyte antigen-DR (HLA-DR)-positive cells were selected on a CD16 versus HLA-DR plot (P3), thereby excluding residual neutrophils and natural killer cells. Subsequently, HLA-DR high/CD14 low cells, representing potentially contaminating B lymphocytes, were excluded using a CD14 versus HLA-DR plot. Because B-cell contamination varies between samples [12], this gating step was consistently applied to all specimens to exclude potential contaminating events. The final monocyte population was then displayed on a CD14 versus CD16 contour plot. Based on the differential expression of CD14 and CD16, monocytes were classified according to the current consensus nomenclature into classical (CD14++CD16−), intermediate (CD14++CD16+), and non-classical (CD14+CD16++) subsets. Using the rectangular gate icon in the graphical interface, we created an analysis gate that encompasses the classic monocyte cluster, which shows high CD14 and low CD16 expression. Then, the gate is optimized so that it includes all cells remaining on the left side and creates a balanced distribution to the right and left of the median. We drew a new rectangular gate to select the cells located to the right of the classical monocyte gate and designated this as the intermediate population. The lower boundary of the gate was aligned with the lower edge of the concentric density rings within the classical monocyte cluster to exclude non-classical cells. Using the bottom edge of the intermediate population gate as a reference, we opened a new rectangular area downward and captured the non-classical monocyte subset by including all cells in this gate. The gating strategy was based on the standardized whole-blood flow cytometry protocol described by Marimuthu et al. [12]. To ensure methodological consistency, all gating procedures and monocyte subset classifications were performed by an experienced biochemical specialist.

### 2.6. Statistical Analysis

The normality of the distribution of continuous variables was assessed using the Shapiro–Wilk test and visual analysis of histograms. Normally distributed continuous variables were expressed as mean ± standard deviation, whereas non-normally distributed variables were presented as median (interquartile range, IQR). Categorical variables were expressed as frequencies and percentages. Categorical variables were compared using the Chi-square test or Fisher’s exact test when appropriate. Mann–Whitney U or Student’s *t* test was utilized to compare two independent groups according to the normal distribution fit. The relationships between variables were evaluated using Spearman correlation analyses. Multivariable binary logistic regression analysis was performed using the Enter method to identify factors independently associated with significant CAS. Age and sex were included irrespective of statistical significance as established confounders, whereas the remaining variables were selected according to statistically significant univariate associations while considering the limited sample size and the risk of overfitting. Two models were constructed: Model 1 included non-classical monocyte percentage, age, sex, and systolic blood pressure, whereas Model 2 included non-classical monocyte percentage together with HbA1c, neutrophil count, and fibrinogen. Model performance was assessed using the Omnibus test of model coefficients (*p* < 0.05, indicating overall model significance), Nagelkerke’s R^2^, and the Hosmer–Lemeshow goodness-of-fit test (*p* > 0.05, indicating good model fit). Multicollinearity among the independent variables was evaluated using variance inflation factors (VIFs) in Jamovi software (version 2.7.38). Only variables with VIF values < 3 were included in the multivariable logistic regression models. All statistical evaluations were performed using IBM SPSS version 26.0 (IBM Corp., Armonk, NY) and MedCalc version 23.4.5 (MedCalc Software Ltd., Ostend, Belgium). Statistical significance was set at *p* < 0.05.

## 3. Results

A total of 73 participants were included in the study: 44 patients with CAS < 50% and 29 patients with CAS ≥ 50%. Demographic and clinical characteristics of the study population are presented in Table 1. Patients with CAS ≥ 50% exhibited significantly higher mean cIMT values and systolic blood pressure than those with CAS < 50%. Although patients with significant stenosis tended to be older and had a higher prevalence of previous stroke, these differences were not statistically significant (*p* = 0.078 and *p* = 0.052, respectively). No significant differences were observed between the groups in terms of sex, BMI, waist circumference, smoking status, alcohol consumption, diabetes mellitus, hypertension, coronary artery disease, or medication use (all *p* > 0.05).

The biochemical and monocyte subset findings according to stenosis are summarized in Table 2. Patients with CAS ≥ 50% had significantly higher classical monocyte percentages than those with CAS < 50% [82.6 (78.6–86.8) vs. 79.5 (77.0–83.6), *p* = 0.044]. Non-classical monocyte percentages were significantly lower in patients with CAS ≥ 50% than in those with CAS < 50% (9.89 ± 3.89% vs. 12.1 ± 4.12%, *p* = 0.027), with a moderate effect size (Cohen’s d = 0.55). Intermediate monocyte percentages were similar between the groups (*p* = 0.199). Inflammatory parameters, including white blood cell count (WBC), neutrophil count, SIRI, and fibrinogen levels, were significantly higher in patients with CAS ≥ 50% than in those with CAS < 50%. HbA1c levels were moderately but significantly increased in patients with significant stenosis. Patients with CAS ≥50% had significantly lower LDL-C and HDL-C levels than those with CAS < 50%. No significant differences were observed in MCP-1, lymphocyte count, monocyte count, platelet count, NLR, MLR, PLR, SII, glucose, creatinine, CRP, albumin, ALT, GGT, triglycerides, non-HDL-C, and remnant cholesterol levels.

The significant correlations between monocyte subsets and clinical or laboratory parameters are presented in Table 3. The classical monocyte percentages demonstrated weak positive correlations with age and NLR, whereas a weak inverse correlation was observed with LDL-C level. Non-classical monocyte percentages showed significant negative correlations with several inflammatory markers including neutrophil count, fibrinogen level, NLR, and SII. Non-classical monocytes were also inversely correlated with cIMT and HbA1c levels. Conversely, positive correlations were observed between non-classical monocytes and diastolic blood pressure, hemoglobin, and HDL-C levels.

Significant correlations between the average cIMT and clinical and laboratory parameters are presented in Table 4. The average cIMT showed a significant negative correlation with non-classical monocyte percentages, hemoglobin levels, and HDL-C levels. In contrast, positive correlations were observed between the average cIMT and creatinine, fibrinogen, neutrophil count, triglyceride levels, and SIRI.

Multivariable logistic regression analyses were performed to identify variables that were independently associated with significant CAS (Table 5). Model 1 demonstrated a significant overall fit (Omnibus test of model coefficients, *p* = 0.018), explained 20.4% of the variance (Nagelkerke’s R^2^ = 0.204), and showed acceptable goodness of fit (Hosmer–Lemeshow test, *p* = 0.247). After adjusting for age, sex, and systolic blood pressure, the non-classical monocyte percentage remained independently associated with significant CAS (OR 0.872, 95% CI 0.765–0.994; *p* = 0.040). Model 2 also demonstrated a significant overall fit (Omnibus test of model coefficients, *p* = 0.004), explained 25.4% of the variance (Nagelkerke’s R^2^ = 0.254), and showed acceptable goodness of fit (Hosmer–Lemeshow test, *p* = 0.206). In this model, fibrinogen remained independently associated with significant CAS (OR 1.008, 95% CI 1.001–1.015; *p* = 0.032), whereas the association between non-classical monocyte percentage and significant CAS was no longer statistically significant (*p* = 0.054).

## 4. Discussion

The present study demonstrated differences in monocyte subset distribution according to CAS severity. Patients with significant CAS (≥50%) had higher classical monocyte percentages and lower non-classical monocyte percentages than those with non-significant stenosis, whereas intermediate monocyte proportions were similar between the groups. In addition, classical monocyte percentages showed a weak positive correlation with NLR, whereas non-classical monocyte percentages were inversely correlated with cIMT and several inflammatory and metabolic parameters, including fibrinogen, neutrophil count, NLR, SII, and HbA1c. Although lower non-classical monocyte percentages were associated with significant CAS after adjustment for demographic variables, this association was attenuated after further adjustment for fibrinogen, neutrophil count, and HbA1c, whereas fibrinogen remained independently associated with significant stenosis. Although the underlying mechanisms cannot be determined from this cross-sectional analysis, these findings may suggest a potential relationship between monocyte subset distribution and the inflammatory processes accompanying advanced carotid atherosclerosis.

The observed elevation in classical monocytes in patients with significant CAS aligns with the results of several recent studies. Cifani et al. [13] found significantly increased classical monocytes in acute aortic dissection compared to CAS and risk factor controls, suggesting that classical monocyte expansion may reflect acute inflammatory activation in vascular pathology. However, a meta-analysis by Oh et al. [14] presented a contrasting pattern, reporting lower percentages of classical monocytes in subjects with cardiometabolic disorders than in healthy controls (SMD = −1.21; 95% CI: −1.92, −0.50; *p* = 0.0009). This apparent discrepancy likely reflects methodological heterogeneity across studies, including differences in disease phenotypes (acute versus chronic atherosclerosis), patient populations (coronary versus carotid disease), and analytical approaches (absolute counts versus relative percentages). The focus of the present study on carotid stenosis severity, rather than a comparison with healthy controls, may explain the observed classical monocyte elevation in advanced disease. The mechanistic basis for classical monocyte involvement in atherosclerosis is well established. Classical monocytes (CD14++CD16−) express high levels of CCR2, the receptor for MCP-1/CCL2, which facilitates their recruitment to inflamed arterial walls [15]. Sbrana et al. [10] demonstrated that circulating classical monocytes (Mon1) in stable coronary artery disease exhibit elevated expression of CCR2, CCR5, and CX3CR1, with these receptor patterns correlating with plaque calcification metrics. This receptor profile supports a model in which classical monocytes are preferentially recruited to atherosclerotic lesions.

The lower proportion of non-classical monocytes observed in patients with significant CAS contrasts with several reports linking elevated non-classical monocytes to adverse cardiovascular outcomes. Feinstein et al. [16] reported that non-classical monocytes were associated with cIMT progression in a Multi-Ethnic Study of Atherosclerosis cohort. Similarly, Abo-Aly et al. [17] found that higher baseline CD14+CD16++ monocyte counts predicted worse composite outcomes after ST-elevation myocardial infarction (STEMI). Tomas et al. [18] characterized non-classical monocytes as endothelial “patrolling” cells that migrate via CX3CR1-dependent pathways to detect and clear damaged cells and debris, suggesting potential atheroprotective functions. Marsh et al. [19] demonstrated the rapid depletion of circulating non-classical monocytes after coronary reperfusion in STEMI, suggesting their recruitment to the injured myocardium and highlighting a dual context-dependent pro-inflammatory role despite their homeostatic patrolling functions. The apparent discrepancy between our findings and those of previous studies may reflect the considerable heterogeneity among studies evaluating monocyte subsets in cardiovascular disease. Recent reviews have emphasized that differences in patient populations, disease stage, flow cytometric gating strategies, and assessment of relative versus absolute monocyte subset counts may substantially influence study findings. Furthermore, advances in high-parameter flow cytometry and single-cell RNA sequencing have demonstrated that conventional CD14/CD16-based classification does not fully capture the biological heterogeneity of circulating monocytes, identifying additional phenotypically and functionally distinct subpopulations beyond the traditional three-subset model. These methodological and biological differences may partly account for the inconsistent findings reported across studies [20,21]. Feinstein et al. [16] found a sex-specific association in which non-classical monocytes predicted cIMT progression in men but not in women. In the current study, sample size constraints precluded us from stratifying the analyses by sex, an important area for future research. Hormonal effects on monocyte subset distribution and function may be involved in sex-specific atherosclerotic risk profiles. Importantly, the total absolute monocyte counts were comparable between the study groups. Therefore, the observed lower relative proportion of non-classical monocytes should be interpreted alongside the corresponding increase in the proportion of classical monocytes, as monocyte subsets were assessed as relative percentages and were inherently compositional.

Although intermediate monocyte percentages did not differ significantly between the CAS severity groups in the present study, previous studies have implicated this subset in atherosclerosis [14,22,23]. SahBandar et al. [22] reported a significant association between intermediate monocyte counts and carotid IMT, while a meta-analysis by Oh et al. [14] found higher intermediate monocyte percentages in individuals with cardiometabolic disorders. Intermediate monocytes (CD14++CD16+) are considered to exhibit a transitional phenotype with enhanced inflammatory cytokine production [23]. The lack of significant intermediate monocyte differences in the present study may reflect the cross-sectional design and focus on established stenosis rather than on early atherosclerotic changes. Intermediate monocytes may play a more prominent role in the transition from subclinical to clinical atherosclerosis, as suggested by their strong correlation with cIMT in the SahBandar study [22].

The absence of significant MCP-1 differences between the CAS severity groups in the present study contrasts with substantial evidence linking this chemokine to atherosclerotic disease progression. Georgakis et al. [24] demonstrated that plaque MCP-1 concentrations were associated with histological features of plaque vulnerability, suggesting that local plaque expression may be more relevant to disease severity than circulating levels. Similarly, studies evaluating circulating MCP-1 have reported associations between subclinical carotid atherosclerosis and cIMT progression [25,26,27]. Several factors related to these positive associations may account for the null finding of the present study. The present study compared patients with different degrees of established stenosis, whereas studies reporting positive associations have often compared patients with subclinical atherosclerosis with those without disease. The timing of MCP-1 measurement relative to plaque evolution may be important; MCP-1 may be most elevated during active plaque progression rather than in stable established stenosis. Consistent with the central role of MCP-1 in monocyte recruitment, several interventional studies have evaluated whether this pathway can be therapeutically modulated. Makarewicz-Wujec et al. [28] demonstrated that intensive dietary intervention reduced plasma MCP-1 levels and altered plaque composition, indicating that lifestyle modifications can modulate monocyte recruitment pathways. Carvalho et al. [29] reported that despite 6 months of lipid-lowering and antiplatelet therapy after acute myocardial infarction, patients exhibited persistent pro-inflammatory monocyte phenotypes, including higher percentages of classical monocytes, lower percentages of non-classical monocytes, and altered chemokine receptor expression. This persistence of inflammatory monocyte phenotypes despite standard medical therapy suggests that current treatments do not completely address monocyte-driven inflammation. Novel therapeutic approaches targeting monocyte recruitment and activation are currently under investigation. CCR2 antagonists, which block MCP-1-mediated monocyte recruitment, have shown promise in preclinical studies but have faced challenges in clinical translation [30].

In the present study, fibrinogen levels were significantly higher in patients with significant CAS, consistent with its established role as both a coagulation factor and an inflammatory mediator. Zhang et al. [31] further demonstrated that circulating CD14++CD16+ intermediate monocytes and fibrinogen synergistically predicted cardiovascular events after myocardial infarction. This interaction may be mediated by the expression of integrin αMβ2 (Mac-1) and protease-activated receptors (PAR-1 and PAR-3) on monocyte subsets, which facilitates fibrinogen-dependent inflammatory responses [31]. Consistent with these observations, fibrinogen remained independently associated with significant CAS in our exploratory multivariable model, whereas the association between non-classical monocyte percentage and stenosis severity was no longer statistically significant after adjusting for fibrinogen, neutrophil count, and HbA1c. We also observed significantly higher NLR, MLR, and SIRI values in patients with significant CAS. As SIRI integrates neutrophil, monocyte, and lymphocyte counts, it may better reflect the overall inflammatory status than individual leukocyte populations. Similarly, Afanasieva et al. [32] reported that monocyte-based inflammatory indices were associated with adverse cardiovascular outcomes, supporting their potential value as markers of vascular inflammation. These associations may reflect the coordinated involvement of innate and adaptive immune responses during atherosclerosis [33]. The integration of these parameters into composite indices may capture the overall inflammatory burden more effectively than individual cell counts.

The present study found significantly lower LDL-C and HDL-C levels in patients with significant CAS. However, the proportion of patients receiving lipid-lowering therapy did not differ significantly between the study groups. Therefore, the observed differences in lipid levels cannot be solely attributed to lipid-lowering therapy. Other factors, including differences in treatment dose or duration, medication adherence, lifestyle modifications, and additional clinical characteristics not evaluated in the present study, may also have influenced the observed lipid profile. Oxidized LDL (oxLDL) is a potent activator of monocytes and macrophages that promotes foam cell formation and inflammatory cytokine production [34]. Although the present study measured total LDL-C rather than oxLDL, the relationship between lipid levels and monocyte phenotypes in the context of statin therapy represents an important area for future investigation.

AlMarri et al. [35] developed a Monocyte Atherosclerotic Risk Score (MARS) incorporating monocyte-platelet aggregates and CD14+/CD16+ monocytes, which correlated more closely with cIMT. MARS was highly predictive of cIMT-determined high cardiovascular risk and the presence of carotid plaque, demonstrating superior performance compared to traditional risk algorithms [35]. These findings indicate that monocyte subset profiling may complement conventional cardiovascular risk assessments. However, several challenges remain before its clinical implementation. Although the differences in monocyte subset proportions observed in our study were statistically significant, their magnitude was modest. Therefore, our findings do not support the routine clinical use of flow cytometric monocyte subset analysis in patients with CAS. In addition to requiring validation in larger prospective multicenter studies, practical limitations, including specialized instrumentation, standardized gating strategies, experienced personnel, higher costs, and limited inter-laboratory standardization, currently restrict its routine application. Furthermore, because only relative monocyte subset proportions were evaluated, future studies integrating both relative and absolute monocyte counts are needed to better define their clinical utility and determine whether they provide incremental value beyond established clinical, imaging, and laboratory markers.

This study has several limitations that warrant consideration. First, the cross-sectional design precludes causal inference; longitudinal studies are needed to determine whether monocyte subset changes precede or follow atherosclerotic progression. Second, the sample size (n = 73) limited the statistical power for subgroup analyses, particularly sex-stratified analyses. Third, this study assessed circulating monocyte subsets and MCP-1, but not tissue-resident monocytes/macrophages in atherosclerotic plaques. Future studies integrating circulating and tissue compartments would provide more complete mechanistic insights. In addition, monocyte subsets were reported as percentages rather than as directly measured absolute counts. Because bead-based or volumetric flow cytometric methods were not used, absolute monocyte subset counts were unavailable. Fourth, the study population consisted of patients undergoing carotid ultrasound evaluation, potentially introducing a selection bias. Another limitation of the present study is that only symptomatic patients who underwent carotid Doppler ultrasonography were included. Future studies including both asymptomatic and symptomatic individuals with different degrees of CAS may provide a more comprehensive understanding of monocyte subset alterations throughout the carotid atherosclerosis spectrum. Another limitation of this study is that plaque vulnerability was not evaluated using standardized ultrasonographic methods, including the Gray–Weale classification, gray-scale median normalization, or the assessment of intraplaque hemorrhage, fibrous cap status, and neovascularization. Therefore, plaque stability could not be assessed comprehensively. Furthermore, cIMT was measured manually using electronic calipers rather than an automated software, and intra-/inter-observer reproducibility was not assessed. Fifth, the study measured total MCP-1 levels but not other chemokines (CX3CL1 and CCL5) that regulate monocyte trafficking; comprehensive chemokine profiling may reveal additional associations with stenosis severity. Finally, no formal adjustment for multiple comparisons was applied because the primary objective of this study was to evaluate the association between circulating monocyte subsets and CAS severity. The remaining biochemical, inflammatory, and correlation analyses were considered as secondary and exploratory analyses. Therefore, findings with borderline statistical significance should be interpreted with caution and confirmed in larger prospective studies. Future studies might extend the evaluation of circulating monocyte subsets beyond carotid artery disease to a more comprehensive assessment of systemic atherosclerosis. As atherosclerosis is a systemic disorder that may manifest differently across individual vascular beds, integrating monocyte subset analysis with complementary vascular assessments, such as ankle-brachial index (ABI), toe-brachial index (TBI), pulse wave velocity (PWV), and established cardiovascular biomarkers (e.g., hs-cTnT and NT-proBNP), may provide a more comprehensive characterization of vascular inflammation and cardiovascular risk [36].

## 5. Conclusions

In conclusion, patients with significant CAS (≥50%) exhibited higher proportions of classical monocytes and lower proportions of non-classical monocytes than those with CAS < 50%. Lower non-classical monocyte percentages were associated with significant CAS and showed inverse correlations with cIMT and inflammatory markers. However, the association between non-classical monocyte percentages and significant CAS was attenuated after adjusting for inflammatory markers. These findings indicate that the observed association between lower non-classical monocyte percentages and stenosis severity was influenced by concomitant inflammatory markers, suggesting that circulating monocyte subset distribution should be interpreted within a broader inflammatory context rather than as an isolated independent determinant of disease severity. Further prospective studies are needed to clarify the causal relationships and clinical significance of these associations.

## Figures and Tables

**Table 1:** Demographic and clinical characteristics according to carotid artery stenosis category.

Variables	CAS < 50% (n = 44)	CAS ≥ 50% (n = 29)	*p*-Value
Age (years)	65 ± 10	69 ± 9	0.078 ^a^
Sex, n (%)			
Female	27 (61.4)	16 (55.2)	0.777 ^b^
Male	17 (38.6)	13 (44.8)	
BMI (kg/m^2^)	28.3 ± 4.10	27.9 ± 3.94	0.651 ^a^
Waist circumference (cm)	97.2 ± 9.82	100 ± 6.70	0.181 ^a^
Mean cIMT (mm)	0.85 (0.75–1.04)	1.10 (0.89–1.20)	**0.002** ^c^
Systolic BP (mmHg)	136 ± 16.6	146 ± 21.5	**0.030** ^a^
Diastolic BP (mmHg)	79.9 ± 10.4	78.0 ± 10.1	0.442 ^a^
Alcohol consumption, n (%)	5 (11.4)	6 (20.7)	0.327 ^b^
Smoking, n (%)	16 (36.4)	15 (51.7)	0.290 ^b^
DM, n (%)	14 (31.8)	14 (48.3)	0.242 ^b^
Hypertension, n (%)	28 (63.6)	24 (82.8)	0.133 ^b^
CAD, n (%)	11 (25.0)	7 (24.1)	1.000 ^b^
Previous stroke, n (%)	2 (4.50)	6 (20.7)	0.052 ^b^
ACE inhibitors/ARBs, n (%)	17 (38.6)	10 (34.5)	0.911 ^b^
Beta-blockers, n (%)	10 (22.7)	8 (27.6)	0.846 ^b^
CCB, n (%)	5 (11.4)	1 (3.40)	0.392 ^b^
Diuretics, n (%)	1 (2.30)	3 (10.3)	0.294 ^b^
Oral antidiabetic agents, n (%)	13 (29.5)	9 (31.0)	1.000 ^b^
Insulin therapy, n (%)	1 (2.30)	4 (13.8)	0.077 ^b^
Lipid-lowering agents, n (%)	5 (11.4)	6 (20.7)	0.327 ^b^

Those written in bold are statistically significant (*p* < 0.05). Data are presented as mean ± standard deviation, median (25–75th percentiles), or number (percentage), as appropriate. ^a^: Student’s *t*-test; ^b^: Chi-square test; ^c^: Mann–Whitney U test. CAS: carotid artery stenosis; BMI: body mass index; cIMT: carotid intima-media thickness; BP: blood pressure; DM: diabetes mellitus; CAD: coronary artery disease; ACE inhibitors/ARBs: angiotensin-converting enzyme inhibitors/angiotensin receptor blockers; CCB: calcium channel blockers.

**Table 2:** Monocyte subsets and biochemical variables according to carotid artery stenosis category.

Variables	CAS < 50% (n = 44)	CAS ≥ 50% (n = 29)	*p*-Value
Classical monocytes (%)	79.5 (77.0–83.6)	82.6 (78.6–86.8)	**0.044** ^a^
Intermediate monocytes (%)	6.03 (3.31–9.24)	4.73 (2.92–6.96)	0.199 ^a^
Non-classical monocytes (%)	12.1 ± 4.12	9.89 ± 3.89	**0.027** ^b^
MCP-1 (pg/mL)	169 (114–388)	165 (76.7–334)	0.279 ^a^
WBC (×10^9^/L)	6.48 (5.49–8.05)	7.62 (6.30–9.85)	**0.021** ^a^
Neutrophil count (×10^9^/L)	3.96 (2.98–4.89)	4.78 (3.59–6.38)	**0.027** ^a^
Lymphocyte count (×10^9^/L)	1.91 (1.45–2.58)	2.00 (1.55–2.77)	0.481 ^a^
Monocyte count (×10^9^/L)	0.41 (0.35–0.52)	0.50 (0.35–0.60)	0.131 ^a^
Platelet count (×10^9^/L)	258 (212–301)	277 (225–326)	0.182 ^a^
HGB (g/dL)	13.7 (12.9–14.8)	13.6 (12.3–14.3)	0.370 ^a^
NLR	1.88 (1.55–2.38)	2.20 (1.74–3.25)	0.155 ^a^
MLR	0.20 (0.18–0.27)	0.23 (0.20–0.30)	0.404 ^a^
PLR	133 (104–160)	121 (105–161)	0.744 ^a^
SIRI	0.81 (0.58–1.28)	1.29 (0.84–1.58)	**0.036** ^a^
SII	469 (392–683)	545 (439–890)	0.091 ^a^
Glucose (mg/dL)	110 (100–124)	111 (99.0–149)	0.502 ^a^
Creatinine (mg/dL)	0.82 (0.70–0.93)	0.80 (0.73–1.07)	0.460 ^a^
Fibrinogen (mg/dL)	367 ± 74.2	423 ± 97.9	**0.007** ^b^
CRP (mg/L)	1.58 (1.07–4.09)	2.52 (1.08–5.92)	0.427 ^a^
HbA1c (%)	6.00 (5.60–6.30)	6.20 (5.90–7.00)	**0.049** ^a^
Albumin (g/L)	46.0 ± 2.56	45.1 ± 3.13	0.199 ^b^
ALT (IU/L)	17.8 (12.8–23.2)	14.0 (11.9–17.7)	0.068 ^a^
GGT (IU/L)	19.5 (14.5–27.5)	20.0 (14.0–26.3)	0.955 ^a^
TC (mg/dL)	205 ± 38.3	188 ± 40.0	0.073 ^b^
Triglyceride (mg/dL)	121 (102–149)	134 (102–164)	0.395 ^a^
LDL-C (mg/dL)	133 ± 35.1	114 ± 29.6	**0.021** ^b^
HDL-C (mg/dL)	50.9 (45.9–60.8)	42.2 (36.8–60.2)	**0.023** ^a^
Non-HDL-C (mg/dL)	152 ± 39.1	140 ± 37.3	0.194 ^b^
Remnant-C (mg/dL)	17.4 (11.6–25.7)	20.0 (12.8–31.9)	0.232 ^a^

Those written in bold are statistically significant (*p* < 0.05). ^a^: Mann–Whitney U test; ^b^: Student’s *t*-test. CAS: carotid artery stenosis; MCP-1: monocyte chemoattractant protein-1; WBC: white blood cell; HGB: hemoglobin; NLR: neutrophil-to-lymphocyte ratio; MLR: monocyte-to-lymphocyte ratio; PLR: platelet-to-lymphocyte ratio; SIRI: systemic inflammation response index; SII: systemic immune-inflammation index; CRP: C-reactive protein; HbA1c: hemoglobin A1c; ALT: alanine aminotransferase; GGT: gamma-glutamyl transferase; TC: total cholesterol; LDL-C: low-density lipoprotein cholesterol; HDL-C: high-density lipoprotein cholesterol; Non-HDL-C: non-high-density lipoprotein cholesterol; Remnant-C: remnant cholesterol.

**Table 3:** Significant correlations between monocyte subsets and clinical, biochemical parameters.

**Variables**	**Classical Monocytes** **(r)**	***p*** **-Value**
Age (years)	0.240	0.041
LDL-C (mg/dL)	−0.244	0.037
NLR	0.233	0.047
**Variables**	**Non-Classical Monocytes (r)**	***p*** **-Value**
Diastolic BP (mmHg)	0.360	0.002
Mean cIMT (mm)	−0.320	0.006
Fibrinogen (mg/dL)	−0.254	0.030
Neutrophil count (×10^9^/L)	−0.423	<0.001
HGB (g/dL)	0.293	0.012
NLR	−0.393	0.001
SII	−0.402	<0.001
HbA1c (%)	−0.287	0.014
HDL-C (mg/dL)	0.252	0.032

Data are presented as Spearman’s correlation coefficients (r) and *p*-values. LDL-C: low-density lipoprotein cholesterol; NLR: neutrophil-to-lymphocyte ratio; BP: blood pressure; cIMT: carotid intima-media thickness; HGB: hemoglobin; SII: systemic immune-inflammation index; HbA1c: hemoglobin A1c; HDL-C: high-density lipoprotein cholesterol.

**Table 4:** Significant correlations between average cIMT and clinical, biochemical parameters.

Variables	Average cIMT (r)	*p*-Value
Non-classical monocytes (%)	−0.320	0.006
Creatinine (mg/dL)	0.284	0.015
Fibrinogen (mg/dL)	0.268	0.022
Neutrophil count (×10^9^/L)	0.262	0.025
HGB (g/dL)	−0.253	0.031
HDL-C (mg/dL)	−0.274	0.019
Triglyceride (mg/dL)	0.239	0.042
SIRI	0.264	0.024

cIMT: carotid intima-media thickness; HGB: hemoglobin; HDL-C: high-density lipoprotein cholesterol; SIRI: systemic inflammation response index.

**Table 5:** Multivariable logistic regression analyses for significant carotid artery stenosis (CAS ≥ 50%).

**Model 1**					
**Variables**	**B**	**S.E.**	**Wald**	***p*** **-Value**	**Adjusted OR (95% CI)**
Non-classical monocytes (%)	−0.137	0.067	4.230	**0.040**	0.872 (0.765–0.994)
Age (years)	0.029	0.031	0.878	0.349	1.029 (0.969–1.093)
Systolic BP (mmHg)	0.029	0.016	3.555	0.059	1.030 (0.999–1.062)
Sex (Male)	0.438	0.544	0.647	0.421	1.549 (0.533–4.501)
**Model 2**					
**Variables**	**B**	**S.E.**	**Wald**	***p*** **-Value**	**Adjusted OR (95% CI)**
Non-classical monocytes (%)	−0.064	0.073	0.763	0.383	0.938 (0.812–1.083)
HbA1c (%)	0.477	0.292	2.665	0.103	1.612 (0.909–2.860)
Neutrophil count (×10^9^/L)	0.088	0.174	0.259	0.611	1.092 (0.777–1.536)
Fibrinogen (mg/dL)	0.007	0.003	4.610	**0.032**	1.008 (1.001–1.014)

The model fit statistics were as follows: Model 1—Omnibus test of model coefficients (*p* = 0.018), Nagelkerke R^2^ = 0.204, and Hosmer–Lemeshow goodness-of-fit test (*p* = 0.247). Model 2—Omnibus test of model coefficients (*p* = 0.004), Nagelkerke R^2^ = 0.254, and Hosmer–Lemeshow goodness-of-fit test (*p* = 0.206). Those written in bold are statistically significant (*p* < 0.05). B: regression coefficient; S.E.: standard error; OR: odds ratio; CI: confidence interval; BP: blood pressure; HbA1c: hemoglobin A1c.

## Data Availability

The original contributions presented in this study are included in the article. Further inquiries can be directed to the corresponding author.

## Appendix A

After the addition of fluorochrome-conjugated antibodies, the samples were vortexed and incubated for 20 min in the dark environment at room temperature (25 °C). Following incubation, 2 mL (1:10 dilution) of lysis solution was added. The samples were vortexed and incubated for 10 min in a dark environment at room temperature. The samples were then centrifuged at 1800 rpm for 5 min, and the supernatant was discarded. Subsequently, 2 mL of CellWash solution was added. The pellet was vortexed and centrifuged at 1800 rpm for 5 min. After removing the supernatant, the cell pellet was resuspended in 350 μL of FACSFlow sheath fluid, vortexed, and immediately analyzed by flow cytometry.
